# The role of alcohol in constructing gender & class identities among young women in the age of social media

**DOI:** 10.1016/j.drugpo.2018.04.009

**Published:** 2018-08

**Authors:** Jemma Lennox, Carol Emslie, Helen Sweeting, Antonia Lyons

**Affiliations:** a MRC/CSO Social & Public Health Sciences Unit, University of Glasgow, UK; b School of Health & Life Sciences, Glasgow Caledonian University, UK; c School of Health, Victoria University of Wellington, Wellington, New Zealand

**Keywords:** Gender, Drinking, Facebook, Femininities, Online, Digital

## Abstract

Research suggests young women view drinking as a pleasurable aspect of their social lives but that they face challenges in engaging in a traditionally ‘masculine’ behaviour whilst maintaining a desirable ‘femininity’. Social network sites such as Facebook make socialising visible to a wide audience. This paper explores how young people discuss young women’s drinking practices, and how young women construct their identities through alcohol consumption and its display on social media. We conducted 21 friendship-based focus groups (both mixed and single sex) with young adults aged 18–29 years and 13 individual interviews with a subset of focus group respondents centred on their Facebook practices. We recruited a purposive sample in Glasgow, Scotland (UK) which included ‘middle class’ (defined as students and those in professional jobs) and ‘working class’ respondents (employed in manual/service sector jobs), who participated in a range of venues in the night time economy. Young women’s discussions revealed a difficult ‘balancing act’ between demonstrating an ‘up for it’ sexy (but not too sexy) femininity through their drinking and appearance, while still retaining control and respectability. This ‘balancing act’ was particularly precarious for working class women, who appeared to be judged more harshly than middle class women both online and offline. While a gendered double standard around appearance and alcohol consumption is not new, a wider online audience can now observe and comment on how women look and behave. Social structures such as gender and social class remain central to the construction of identity both online and offline.

## Introduction

In her important review of the role of alcohol in British women’s lives, Plant (2008) argues that changes in gender roles and in women’s social and economic position, the ‘feminisation’ of the night time economy, sales of cheap alcohol in supermarkets, and female-targeted marketing form the backdrop to increased drinking amongst young women over the past 50 years. A more recent body of qualitative research suggests that young women view alcohol as a pleasurable and important aspect of their social lives (Bancroft, 2012; Guise & Gill, 2007; Seaman & Edgar, 2012), and place value upon sharing drinking and hangover stories (Griffin, Bengry-Howell, Hackley, Mistral, & Szmigin, 2009; Sheehan & Ridge 2001). It is not simply the consumption of alcohol which is considered enjoyable, but also what that consumption represents. This includes peer group popularity and validation resulting from drinking in line with dominant social norms (Emslie, Hunt, & Lyons, 2015; Lunnay, Ward, & Borlagdan, 2011) and the use of alcohol as justification for engaging in traditionally ‘unfeminine’ behaviours such as public rowdiness, with less risk of being viewed as unrespectable than if sober (Griffin, Szmigin, Bengry-Howell, Hackley, & Mistral, 2013; Rúdólfsdóttir & Morgan, 2009).

Despite young women reporting pleasure from their drinking, there remains a gendered double standard around their consumption practices (Atkinson & Sumnall, 2016; de Visser & McDonnell, 2012). There is a disproportionate emphasis on women’s ‘binge’ drinking in the media which is often framed as an attempt to emulate men and as transgressing traditional gender roles (Day, Gough, & McFadden, 2004; Jackson & Tinkler, 2007; Lyons, Dalton, & Hoy, 2006; Measham & Østergaard, 2009; Patterson, Emslie, Mason, Fergie, & Hilton, 2016). While women’s drinking may now be considered socially acceptable, drunk women continue to elicit reactions of visceral repulsion (e.g. ‘skanky’, ‘gross’, ‘trash’, a ‘state’: (MacLean, Pennay, & Room, 2018)); “a ‘*drinking* femininity’ is acceptable but a ‘*drunken* femininity’ is not” (Hutton, Griffin, Lyons, Niland, & McCreanor, 2016) (p85). Anxieties over young women’s drinking and intoxication have also filtered into public health campaigns which suggest that excessive drinking is likely to result in shame and regret (Brown & Gregg, 2012). A ‘vulnerability discourse’ in respect of ‘spoiled’ femininity and (sexual) assault is often used to justify the double standard around gender and intoxication, even by those young people who perceive that heavy drinking by young women shows equality with their male peers (Lyons & Willott, 2008).

Young women can now participate in the (traditionally masculine) culture of intoxication, as long as they display an ‘up for it’ heterosexually attractive femininity (Griffin et al., 2013; Waitt, Jessop, & Gorman-Murray, 2011). The individualism, self-reinvention and consumption inherent in neo-liberalism has led to a ‘postfeminist’ femininity characterised as young, bold, self-confident, sassy and sexual (McRobbie, 2008). However, these individualistic discourses tend to “minimize the role of social structural limitations … in the formation of identities and life paths” (Dobson, 2012), p371 As Dobson (2012) argues, navigating the contradictory discourses of postfeminist femininity is likely to be particularly difficult for young women from lower socio-economic groups, and the risks of failure are also likely to be greater. Our previous work with other authors has focused on the intersections between gender and sexuality (Emslie, Lennox, Ireland, 2017), gender and age (Emslie, Hunt, & Lyons, 2013; Emslie et al., 2015; Lyons, Emslie, & Hunt, 2014; Lyons, McCreanor et al., 2014; Lyons & Willott, 2008; Willott & Lyons, 2012), and gender and ethnicity (Goodwin, Griffin, Lyons, McCreanor, & Moewaka Barnes, 2016) in relation to identity and drinking practices. This paper explores how gender and social class intersect and are embedded in identity and drinking practices.

The importance of the intersection between gender and social class has been highlighted repeatedly. For example, Skeggs (1997) argues that while working class men can use class as a positive source of identity, the description ‘working class’ when applied to women “has been used to signify all that is dirty, dangerous and without value” (p74). It is therefore unsurprising that middle class drinking culture is positioned as ‘normal’, while female working class drinkers in particular, who often lack the economic and cultural resources to consume ‘appropriately’, are pathologised as vulgar, loud, immoral and out of control (Bailey & Griffin, 2017; Brown & Gregg, 2012; Day, Gough, & McFadden, 2003; Haydock, 2014; Skeggs, 1997). This has implications for how women from different class positions drink and participate in the night time economy. A number of studies have found that young women draw on these highly gendered and classed discourses to distance themselves from ‘slutty’, ‘trashy’, ‘tragic’ or ‘chavvy’ (working class) women (Bailey, Griffin, & Shankar, 2015; Griffin et al., 2013; Hutton et al., 2016; Rúdólfsdóttir & Morgan, 2009). Hutton et al. (2016) argue that young women “remain aware of the need to retain control and appear ‘respectable’ (not ‘trashy’); “*positioned othering*” of disreputable others perhaps means that respectability can be maintained” (p82).

Social class, and other axes of social stratification, are further accentuated by venue choice (Forsyth & Lennox, 2010). As Thornton (1995) suggests, different venues “facilitate the congregation of people with like tastes”; club clientele are “pre-sorted and pre-selected” through venue publicity and the door policy which may “refuse admission to those who don’t belong” (p22–24). Working class young women tend to go to ‘mainstream’ venues, which are usually corporately owned, play commercial chart music, have a smart dress code and attract a particular hypersexual performance of femininity: “high heels, short skirts, low-cut tops, fake tan, long, straight and (bottle) blonde hair, smooth bare legs … lots of make-up and a buxom slimness” (Griffin et al., 2013, p194). In contrast, ‘niche’ venues, attract a more middle class or ‘alternative’ clientele, focus on particular music genres and have a more ‘dressed down’ approach to style (Hollands, 2002, Lindsay, 2006). Thus, different venues attract and reinforce different performances of femininity embodied through appearance, style, attitude and consumption choices (Hutton, 2006).

Social media is now an inherent part of many young people’s social lives, identities (boyd, 2014) and drinking practices (Lyons, McCreanor, Goodwin, & Moewaka Barnes, 2017). Young people regularly post and share drinking photographs and engage in ongoing interactions around their drinking practices (McCreanor et al., 2013), while venues in the night time economy employ professional photographers to take pictures of patrons which are then posted on social media to encourage interaction by, for example, ‘liking’, ‘sharing’ and ‘tagging’ (Atkinson & Sumnall, 2016; Goodwin et al., 2016) Social network sites are valued by young people for the pleasure they bring to friendship groups as well as for the ongoing formation of individual and group identities (Lyons, Goodwin, McCreanor, & Griffin, 2015). Facebook, for example, plays a major role in young people’s lives, shaping normative behaviour, enabling the creation of connected identities and promoting (and branding) the self within contemporary cultural worlds (van Dijck, 2013). Yet social media practices are also gendered and classed in ways that go beyond access issues.

Taking and sharing photos and producing online self-displays within drinking cultures have value and provide pleasure for both men and women, but these practices are more challenging for young women. Young women are more invested than young men in their online appearance and identity (Atkinson & Sumnall, 2017; Atkinson & Sumnall, 2016; Lindsay & Supski, 2017, Manago, Graham, Greenfield, & Salimkhan, 2008; Shafie, Nayan, & Osman, 2012) and undertake more work to ensure ‘appropriate’ online displays (Bailey, Steeves, Burkell, & Regan, 2013; Hutton et al., 2016; Lyons, Goodwin, Griffin, McCreanor, & Moewaka Barnes, 2016; Willem, Araüna, Crescenzi, & Tortajada, 2012). While young women have abandoned ‘passive femininity’ online, actively participating in positive ‘girl talk’ and commenting on representations of masculine bodies, ‘highly romanticized heterosexuality’ is still pervasive, making it difficult for non-traditional gender identities to thrive (De Ridder & Van Bauwel, 2013). Other research suggests that online portrayals of drinking cultures are ‘airbrushed’ (Niland et al., 2014); photographs perceived to portray young women as extremely intoxicated or unattractive are removed, untagged or not shared with others (Atkinson & Sumnall, 2016; Brown & Gregg, 2012; Hutton et al., 2016). This pressure to conform to normative expectations around gender is likely to increase, given that social media makes socialising visible to a wider audience and given the rise in ‘shaming’ sites such as ‘Embarrassing Nightclub Photos’ (Lyons, McCreanor et al., 2014).

Online practices around drinking are also highly classed (Dobson, 2014; Goodwin et al., 2016; Hutton et al., 2016) and require different kinds of work and (self) surveillance by users depending on their social location. Maintaining respectability is key for young female drinkers. However, as a number of commentators have recently noted (Atkinson & Sumnall, 2016; Bailey & Griffin, 2017), there is a dearth of research which explores the intersection of gender and class in relation to young people’s drinking and their online social media practices.

The current study aimed to explore these issues among young people in Glasgow, Scotland. This paper examines how young people discuss young women’s drinking practices and their display on social networking sites, focusing on the gendered and classed nature of drinking, online displays and implications for feminine identities.

## Methods

After obtaining ethical approval for the study, the first author conducted 21 focus groups (a total of 91 participants; 46 women) with groups of friends aged between 18 and 29 years, followed by individual Facebook interviews with a sub-sample of 13 focus group participants (7 women) in 2012 and 2013. Friendship groups are a key site where practices around alcohol consumption are adopted (Seaman & Ikegwuonu, 2010) and in which masculine and feminine identities are learned and enacted (Paechter, 2003), and so enabled investigation of the normative understandings that these groups shared and drew upon. The Facebook interviews used the contents of participants’ individual Facebook profiles as prompts for discussion (Livingstone, 2008). This enabled deeper exploration of issues raised in the focus group discussions and the opportunity to discuss any differences between what was discussed in the focus groups and what was displayed on participants’ Facebook pages. During data collection Facebook was in its ‘third phase’ (2008–2013; Brügger, 2015), and included features such as ‘Facebook Places’ (so users could connect posts with their location using geolocation data on mobile devices), chat and video chat on mobile devices, as well as the ‘timeline’ feature introduced at the start of 2012 (chronologically organising the user’s entire Facebook activity) (Brügger, 2015).

A purposive sample of young adults who socialised together was sought for this study. Geographically, the study was limited to Glasgow, Scotland for convenience and to capitalise on the researchers’ knowledge of the city’s clubs and pubs (Forsyth & Lennox, 2010). Sampling criteria for the focus groups were based on characteristics likely to impact on young adults’ gendered identity construction, alcohol use and the type of alcohol-related content shared online. The aim was therefore to recruit both young men and young women, to include those from both middle and working class backgrounds, and those who attended ‘mainstream’ and ‘niche’ venues in the city. Following other studies of young adults (Lindsay 2006; Seaman & Ikegwuonu, 2010), we used current education/employment status as a proxy for social class, recruiting students and those employed in professional jobs to represent middle class respondents and those employed in manual/service sector jobs to represent working class respondents. We also aimed to recruit a subsample of respondents (varying by gender, social class and venue preference) for individual Facebook interviews.

A range of recruitment strategies were employed. Information with a URL link was posted on a local advertising website (Gumtree), personal Twitter and Facebook pages (asking people to share/retweet the study on their own news feeds), the Twitter and Facebook pages of a local nightclub, and on a dedicated Twitter account set up for the study. Posters and flyers were also displayed in university unions, bars, shops and workplaces. In total the Gumtree advert was viewed 1913 times and 41 people contacted the main researcher (28 from the Gumtree advert and 13 from other sources). Potential participants were asked to recruit up to five friends or colleagues to take part in the focus groups, so the composition of groups was determined by the participants themselves.

As shown in Table 1, the final focus group sample consisted of 16 mixed sex groups, three all-female group and two all-male groups; 46 women and 45 men participated in total. Each group consisted of between four and six participants. Only three participants were parents, all were drinkers (reporting drinking between 0 and 120 units in the last week), and all except two men were Facebook users. Subsequently, 13 participants (seven women and six men) recruited from student, professional worker, manual worker and ‘niche’ venue attender focus groups, completed individual Facebook interviews. Female manual workers and mainstream venue attenders could not be recruited for interview due to lack of interest or difficulty arranging a suitable time to meet due to their work or personal commitments.

**Table 1:** Details of focus groups and participants.

Focus group	Group type a	Group composition b	Age range	Alcohol units in past week
1	Students	Mixed (3F 2M)	20–21	20–51
2	Manual workers	Single (4F)	19–25	0–14
3	Students	Mixed (2F 2M)	18–21	0–35
4	Manual workers	Mixed (4F 2M)	22–27	8–31
5	Niche venue attenders	Mixed (2F 2M)	20–24	22–31
6	Professional workers	Single (4F)	25–28	9–22
7	Students	Mixed (1F 3M)	20–22	52–110
8	Students	Single (4F)	20–21	9–31
9	Niche venue attenders	Mixed (3F 1M)	21–23	12–74
10	Professional workers	Mixed (2F 2M)	24–29	19–52
11	Niche venue attenders	Mixed (3F 1M)	All 22	21–63
12	Students	Mixed (1F 4M)	18–21	17–25
13	Manual workers	Single (4M)	23–27	7–48
14	Manual workers	Single (5M)	23–29	42–120
15	Mainstream venue attenders	Mixed (1F 3M)	18–26	0–48
16	Mainstream venue attenders	Mixed (2F 3M)	19–27	1–50
17	Professional workers	Mixed (1F 3M)	22–29	21–50
18	Student Group	Mixed (1F 3M)	18–23	0–40
19	Niche venue attenders	Mixed (1F 3M)	23–27	6–40
20	Bar Workers	Mixed (4F 1M)	21–29	2–27
21	Catering Workers	Mixed (3F 1M)	21–25	14–45

a These categorisations should be considered as giving a ‘flavour’ of the group as a whole; some could have been categorised in multiple ways (e.g. niche venue attenders but also containing some university students).

b F female; M male.

All focus groups and interviews were conducted within the University of Glasgow. Respondents were provided with information about the study and the opportunity to ask any questions, before giving written informed consent. Focus group participants first completed a brief questionnaire about current employment, Facebook use, venue preference and alcohol consumption in the last week. The focus group discussions were semi-structured and included questions about drinking behaviour, men’s and women’s drinking (including discussion of images of intoxicated men and women, and of men and women drinking different beverages), Facebook use, venue preference and experiences of online venue marketing. For the individual Facebook interviews, participants were asked to log on to their Facebook profile and navigate around it, explain the stories behind profile pictures and other photographs (including those removed/not uploaded), discuss their use of status updates about drinking occasions or nights out or ‘checking in’ to drinking venues, and describe their interactions with any bars and clubs on Facebook. Discussions were audio taped and transcribed verbatim. Respondents were provided with food and non-alcoholic drinks and a small payment (in the form of a high street voucher) to thank them for their time. All participant names used here are pseudonyms, and the names of specific venues have been removed.

## Analysis

Transcripts and written notes from the focus groups and individual interviews were read repeatedly and coded within Nvivo. Thematic analysis was used, due to its flexibility and ability to provide a detailed and complex analysis of the data (Braun & Clarke, 2006). Initial coding was broad, inclusive and linked to the research questions and the topic guides (e.g. ‘gender and product choice’; ‘photos shared on Facebook’). These were then broken into sub codes using the tree node function of NVivo (e.g. ‘gender and product choice’ was broken down into sub codes such as ‘masculinities’, ‘femininities’, ‘context’). Finally, codes were sorted and combined into potential overarching themes (e.g. how social class cross-cuts gender and alcohol related behaviour; how gender influences on-line presentations of the self which then influences drinking behaviour in public spaces).

## Findings

The accounts of these young adults show how young women attempted to negotiate the fine line between demonstrating an ‘up for it’ heterosexually attractive femininity while taking care to appear to remain in control (of their drinking and appearance). First we describe how young women negotiated gender identities around alcohol in both public spaces and online displays. Secondly, we explore how the structural constraints of social class made this balancing act harder to maintain for working class women.

## Negotiating gender identities around alcohol and intoxication

### Drunkenness and (Self) surveillance In public spaces

Both young women and young men described women’s drunkenness as being more shocking and unacceptable than men’s. Young women were judged particularly harshly when observed drunk in a public place; accounts focused on their appearance (e.g. falling over high heels, short skirts hitched up, state of undress) and linked intoxication to (spoiled) sexual reputation. Respondents used derogatory words such as ‘disgrace’, ‘state’, ‘shame’, ‘disgusting’, and ‘tramp’ to describe intoxicated women in public places, and suggested that women ‘should maintain a bit of decorum’ or at least limit this ‘shameful’ behaviour to private spaces:-

*Allan It’s more shocking … I’d see a guy and think − ‘lad!’ and see a woman I’d think, I don’t know, ‘you tramp’.*

*Steph Yeah, I know, it’s like a woman should maintain a bit of decorum.* (*FG7, mixed sex, university student group*)

*  Patrick (Some women…) they’re happy to be one of the lads, like you’re cool drinking…*

*Holly They’re like ‘I can drink you under the table’ and then they get in that state. That’s what I think girls like that are…*

*Joanne Trying to show off or something, to guys*.

*Holly And then they end up with their skirt underneath their boobs, you know what I mean? You see everything, you know, or they’re lying in their own sick, it’s disgusting.* (*FG16, mixed sex, mainstream venue group*)

*  Diane I*’*ve been bad but I*’*ve never been like I can*’*t move.*

*Lisa Aye, it*’*s a bit of a disgrace.*

*Researcher What do you mean by a disgrace*?

*Lisa Letting yourself get like that. In the middle of the street. At least wait until you get home and collapse. (laughter).* (*FG2, single sex, manual group*)

Young women described strategies to manage potential intoxication while still participating in drinking in public spaces. For example, Rhonda (FG6, single sex, professional group) discussed having separate male and female ‘rounds’ so women could drink at a slower pace than men, while Courtney (FG10, mixed sex, professional group) described choosing weaker drinks when participating in drinking games to avoid throwing up: “*It was like being part of it but also not being part of it to the point that you don*’*t want to be out of control”.*

The gendered double standard around female intoxication was also linked to, and justified by, discourses around women’s greater vulnerability to sexual or physical assault compared to men. Being groped and leered at in nightclubs was seen by women as a common part of going out:-

*Kay All these men … at least 10, 15 years older than us, very desperate, very disgusting, you know, gross men, just appeared from nowhere*….*They were kind of like pushing against us (Kerry: Grinding)…some of them were trying to touch my boobs, and then one of them literally spun me round, grabbed my vagina and then slid his hand up my body…and it does happen a lot.* (*FG9, mixed sex, niche venue group*)

Given these concerns about safety, young women described not travelling home alone and getting a taxi rather than walking. However, new technology and social media were also employed as protection. Young women’s strategies included appearing to be busy with a mobile phone to dissuade men from talking to them and ‘checking in’ to venues on Facebook to alert others to their presence and/or allow others to track their whereabouts:-

*Chloe I don*’*t want some randomer (stranger) to come and talk to me, so I*’*m like (pretending to be) on my phone.* (*FG4, mixed sex, manual group*)

*Tina Well, I use it [Facebook] to kinda tag people, like, in the clubs that we*’*re in. My dad uses it to make sure … he knows where I am … If I say I*’*m on the bus then he knows I*’*m on my way home … so it*’*s kinda a safety measure as well.* (*FG3, mixed sex, college student group*)

While female participants described some venues as *‘meat markets’* (Kay FG9) where women felt on display to men, they were also conscious of being observed by other women. Respondents discussed the *‘silent warfare’* (Steph FG7) and *‘competitive’* (Yasmin FG11) nature of other women’s gazes. Young women were aware that they would be judged as a group when ‘out with the girls’ − and would judge others in the same way − so felt they had to put extra effort into their appearance so they would not let their friends down. Lucy’s excerpt below demonstrates how drinking practices are constructed at a group level and how individuals regulate themselves to align with group gender performances:-

*Lucy If you*’*re going out with all girls you make more effort to get dressed up cause you know … you*’*re gonna be looked at as a group of girls and you*’*re gonna be compared and you know that they*’*re gonna be like dressing up so you*’*ll be like ‘I’m gonna get my nice dress, I*’*ll get my hair done, it*’*s gonna be girls*’. (*FG17, mixed sex, professional workers group*)

Expected performances of femininity differed between mainstream and niche venues. The extracts below illustrate how gender performances and group norms played out at the very local level of individual bars and clubs, and how they worked to ‘other’ those perceived as not belonging to the same community of practice. For example, Holly castigated a woman dressed too casually at a mainstream club (where hypersexual performances of femininity were the norm) for not making ‘an effort’, while Andrea suggested that a woman dressed for a mainstream club would be derided as overdressed and out of place at a niche venue:-

*Holly If I saw a girl come out to the dancing (at mainstream venue) … with a T-shirt on, a pair of black trousers and a pair of flat shoes, you*’*d be like ‘well she could’ve made an effort*’ *… She*’*s at the dancing *….*I don*’*t do it for the guys, but you do it for yourself. *….* I know how I think when I see another girl so I think ‘well if I go out, other girls are gonna think that’, do you know what I mean? (FG16, mixed sex, mainstream venue group)*

*  Andrea Cause if I saw someone who I thought looked like who should be in [Mainstream venue] at [Niche venue] I know that me and my pals would just be like ‘oh look at her, what a state’, so it works both ways. (FG19, mixed sex, niche venue group)*

### Drunkenness and (Self) surveillance online

Surveillance extended online, necessitating a similar but virtual balancing act between portraying a fun-loving, conventionally attractive femininity while still retaining control and respectability. While young women enjoyed sharing drinking stories and having photographs of themselves on Facebook, they were aware of how these images would be judged (by others and by themselves). Photos in which they considered themselves to look overly intoxicated or unattractive would not be uploaded or ‘tagged’, or would be removed. In her individual interview, Courtney (from FG10, professional group) described how she was happy for people to know she went to a mainstream venue recognised for the high level of intoxication of its patrons (thus identifying as someone able to participate in a hedonistic night out), but did not want them to see her actually engaging in this behaviour herself:-

*Courtney Everyone was just looking in different directions (in the photo) and my hair messy kind of thing, and it was just like, it was not a good look and it made me think that like I haven*’*t looked good that night. I must have looked like. a drunk person … It*’*s not something I*’*d be proud of (on my Facebook). … I*’*d happily say to people ‘oh yeah I went to [Mainstream venue]’ but I wouldn*’*t want anyone to see me actually in the picture because it was not a good picture, and it was embarrassing.*

Some young women described specific strategies to show a fun ‘up for it’ online identity without compromising their respectability. These included balancing images of intoxication with photos of other leisure pursuits such as outdoor activities to portray a more ‘rounded’ persona. Others discussed how they assembled their online identities by using carefully posed images where they looked attractive and in control as their profile picture (the most prominent and easily accessible image on Facebook profiles), and only uploading drunk pictures in less obvious positions. For example, in her individual interview, Kerry (from FG9, niche venue group) explained how she chose a flattering profile image (where she looked ‘serene’ and ‘nice) but stated: “*in my regular pictures like yeah, definitely, fun trumps looks”*. Similarly, in her interview, Steph (from FG7, university student group) described posting drunken pictures of herself, providing evidence of active participation in the night time economy to those in her close friendship group. However, by choosing not to “tag” these images, they would be hidden from her wider networks, who only saw images of her displaying a more ‘respectable’ femininity.

*Steph If I think I don*’*t want people to see me looking this drunk, then I will de-tag it or I won*’*t put it up. Or I might have them in the albums … I*’*ve not tagged myself but I*’*ve still put them on-line in the good spirit of the night out … Like this picture, I mean no-one wants that on their profile but it has to go up there [blurry pictures of night out with mixed sex group where everyone looks drunk and sweaty]. So I, yeah, I wouldn*’*t want to put all of them up in association with me. It*’*s fine if they*’*re in an album. If someone takes the time and effort to look through them then they must really wanna see pictures of me.*

Women appeared much more aware than men of the way they were represented online and their narratives suggested the time and care they took ‘curating’ these virtual identities. Steph’s use of the phrase ‘in association with me’ (above) suggests that careful ‘layered’ display of her images allowed her to distance herself from potential criticism regarding ‘appropriate’ feminine appearance and behaviour. Michelle and Chloe (below) also discussed how they did not tag images of themselves looking the ‘worse for wear’ on their profile. This was partly because they did not wish their wider networks to see these images but also because they did not want to be constantly reminded of occasions when they felt they had not looked attractive and in control:-

*Michelle I don*’*t want everybody that*’*s pals with me to see it. Like it*’*s alright if somebody*’*s like scrolling through all their photos and like glimpses past it. But if they come onto my profile and see pictures of me looking a bit worse for wear or something.*

*Chloe It*’*s also you don*’*t want the reminder on your page like all the time.* (*FG4, mixed sex, manual group*)

Finally women were also aware of potential wider audiences. There was a fear of appearing on websites such as ‘embarassingnightclubphotos.com’ which highlights pictures of drunk, undressed clubbers for ridicule by their audience. There was also an awareness that photographs of patrons were used to promote mainstream venues online. For example, Marie described how ‘clubber of the week’ prizes were invariably awarded to young women who most closely embodied the ‘hypersexual femininity’ valued by these venues (i.e. slim young women in low cut, short dresses):-

*Marie [Mainstream venue] also do. “clubber of the week”. Their photographer will pick a photo at random and post it and say ‘if you know these people then tag them in it cause they’ve won … a booth in the VIP (area), and a bottle of champagne*’ *or whatever. Aye. But it*’*s always stick thin girls with their dresses away up here (indicates short) and their chebs (breasts) out.* (*FG4, mixed sex, manual group*)

These promotions and the ubiquity of promotional photographers had implications for women’s gendered performances in mainstream venues. Because they could be widely viewed online by any followers of the venue’s Facebook pages, ‘correct’ feminine performances were required. For example, Lucy described how she adopted a classic feminine pose when approached by a nightclub photographer, rather than the ‘funny girl’ persona she assumed when photographed by friends. This demonstrates how behaviour in public spaces is influenced by an awareness of how this will be represented on social network sites, illustrating the blurring of ‘offline’ and ‘online’ worlds for many young people:-

*Lucy I changed the way I would take a photo [when approached by a nightclub photographer]…because it*’*s not my friends that are gonna view it. It goes on the Facebook for that club…and I*’*m all like [poses] and I*’*m smiling and I*’*m not doing a funny face so I think it does change how I pose ‘cause I don’t want strangers looking at me and writing comments (like) ‘oh, look at the state of her’* (*FG17, mixed sex, professional group*)

## Negotiating class and gender identities around alcohol and intoxication

Both young women and young men appeared to judge the same drinking practices more harshly in women perceived to be working-class than those perceived to be middle class. These accounts were often elicited by the photo prompts used in the focus groups. Nadine, for example, responding to an image of two apparently drunk women, stated that she had no sympathy for them because they were ‘neds’ (a derogatory Scottish term for working class young people, similar to the term ‘chav’ in England). Her extract demonstrates how clothing, shoes and fake tan were read as signifiers of social class:-

*Nadine Looks like pure bams (idiots) and they*’*re all wearing stupid clothes*….*If it was a person who*’*s drank too much I*’*d feel sorry for them – (but) because they*’*re neds I don*’*t care, cause look at that tan…just look at what she*’*s wearing, look at those shoes!* (*FG5, mixed sex, niche venue group*)

Similarly, perceptions of drinking pints of beer or lager (a traditionally ‘masculine’ behaviour) were very different when focused on a photo prompt of two women perceived to be working class compared to a picture of a woman dressed more casually and perceived to be middle class. During discussions in FG17, Clive suggested that the two women perceived to be working class would be rowdy, while the woman perceived to be middle class would be quietly enjoying her drink:-

*Clive I would pre judge these girls if I saw them drinking a pint more than I would her drinking a pint just because of the way they are dressed. … I*’*d make assumptions straight away because of the way they*’*re dressed and I*’*d imagine them to be really loud and like drinking pints but shouting around and stuff whereas I*’*d imagine she just, you know, sitting there enjoying a drink.* (FG17, mixed sex, professional group).

The professional women in FG6 used extremely striking language when contrasting the ‘respectable’ middle class woman with the working class women. Their derogatory words vividly conjured an embodied image (‘council faced’ – presumably referring to the appearance of a working class person living in local authority housing) of a young woman in track suit bottoms consuming pint after pint in the pub:-

*Siobhan Don*’*t think anything of it. I just think it*’*s a girl drinking a pint.*

*Donna She looks quite respectable*.

*Juliet My dad doesn*’*t like it when I*’*m drinking pints ‘cause he’s like ‘oh girls shouldn’t drink a pint*’ *but I*’*m like ‘well, why not?’*

*Donna But then you look respectable, you*’*re not like a ned in a pub with your trackkies* (*track suit bottoms*)*, downing pints*

*Rhonda Not council faced*.

(*FG6, single sex, professional group*)

Young women’s accounts also suggested that social class influenced the ways they prepared for and behaved when drinking in public places, and the meanings attached to these behaviours. This was also reflected in their online presentation of these occasions. Working class women discussed *‘the prep’* (FG4) required for a night out in terms of hair, makeup and outfit selection. The short skirts and high heels which were perceived as obligatory for mainstream venues were not an everyday look for most of these women. In one discussion with shop assistants, Lisa suggested that consuming alcohol before going out helped her to feel more confident about her appearance and less concerned about what others thought of her:-

*Researcher Is it quite important to have a few drinks before you sort of hit the bars and the…?*

*Lisa I think it is aye. It sort of gets the night started and you feel better. Well for me anyway.*

*Researcher In what way do you feel better*?

*Lisa You feel more confident when you*’*re out like dressed up. Like cause you*’*re wearing different clothes so, when you*’*re going out…. You don*’*t usually go out wearing a dress all the time or, with your legs out or whatever. So that*’*s probably, that*’*s what I mean.* (*FG2, single sex, manual group*)

Despite the effort required for this exacting performance of femininity, many young women *‘looked forward’* (Holly FG16) to dressing up for drinking occasions, enjoying the transformation from a more casual appearance restricted by practicalities such as childcare or work uniforms to a more glamorous look. In the focus groups, working class women were more likely than middle class women to discuss their keenness to display the efforts they had put into their appearance by posting images of themselves and their friends dressed up, taken before and during the night out. As the women from FG4 discussed (below), such photos enabled them to display this transformed self on Facebook and they took pleasure in others’ acknowledgement and validation of this authentic self (the *‘real me’)* both online (through ‘likes’ and comments) and in person. Looking back on these glamorous photos also gave these working class young women confidence in their appearance and their ability to perform an appropriate femininity on future occasions:-

*Michelle I like going to work the next day and then people have seen the photos posted of me and going ‘you looked so different! You look really nice in the photos’. I like that sort of feeling.and like ‘well that’s the real me, that*’*s like, it*’*s not the me that you see every single day*’*… I think that*’*s why we take more photos when we*’*re feeling at our best on a night out. It*’*s so that you can sort of like show people that is who you are really.*

*Chloe I think if you are a little bit more confident about yourself you do kinda look good in photos and then the more times you do it, the more better you feel and…it is a little bit more of a confidence booster type thing cause you can look back and go “that was me out and I didn*’*t look terrible so I can go back out and do it all over again”.* (FG4 mixed sex, manual group)

For these working class young women, this traditionally glamorous performance of femininity had value both amongst their immediate circle of friends and among wider social networks:-

*Theresa You don*’*t want to be seen as the funny girl – “aye (yes), she*’*s good for a laugh”. Like, you want somebody to be like “oh she*’*s so pretty, she*’*s so glamorous”.*

(*FG15, mixed sex, mainstream venue group*)

In contrast, middle class women’s narratives suggested that they had a broader range of options. In relation to public spaces, middle class women attended both ‘niche’ and ‘mainstream’ venues in the night time economy, whereas working class women usually only frequented mainstream venues. This range of venues influenced and reflected more variety in acceptable performances of femininity for middle class women. Respondents perceived that ‘niche’ venues focused more on music than appearance. Although image was still important, the femininity performed was casual and lower-key. Similarly, the range of virtual images which young middle class women discussed displaying on Facebook was broader. As Kay explained, funny, rather than glamorous photos had social currency among close friendship groups and were therefore seen as an appropriate way to display an alternative femininity in relation to alcohol use:-

*Kay As a small group we are very comfortable with each other, and actually, if one of us is more inclined to put a ridiculous photo of themselves doing something, like, where they don*’*t look their best or whatever, but they*’*re doing something funny, it*’*s got value to everyone else to see it. we*’*re more likely to all do that and care less about the way we look in photos and more about what the photo tells us.* (*FG9, mixed sex, niche venue group*)

Posting apparently un-posed and casual images on Facebook was perceived by middle class women as an authentic way to display their drinking occasions and to distance themselves from the performances of femininity displayed by working class women. The glamorous online images favoured by working class women (the ‘real me’) were derided as fake, posed images (‘a charade’) by some middle class women. They were particularly scornful of the ‘hall pose’ (usually a photo of a group of women standing in a hallway, dressed in a traditionally feminine way, hand on hip, holding a wine or champagne glass (see also Atkinson & Sumnall, 2016):-

*Lucy My girl mates, we will put up silly drunken photos but I know a lot of girls that just put up the (posed) photos … with the arm on the hip and the glass and they*’*re all glamorous⋯Me and my friends probably do it (put up silly drunken photos) because we want it to look like … we*’*re relaxed and we don*’*t care about our image when really by doing that we do, in a certain way. But I think these girls are the opposite … they*’*re trying to create this glamorous image because they want to know that they*’*re beautiful and they look nice.* (*FG17, mixed sex, professional group*)

*Hazel If there was a girl putting up daft photos all the time and she, had a really good sense of humour, you wouldn*’*t care either. It*’*s people that take themselves seriously and every photo is posed and things. Everyone just finds that funny. They just take the piss out of it and just think well it*’*s just a charade for everyone else really.* (*FG10, mixed sex, professional group*)

However, these often mocking responses from middle class women in the focus groups were complicated by the data collected in the individual interviews. Here, it became evident that middle class women also had traditionally feminine posed pictures on their Facebook profiles, the presence of which required significant discursive work. For example, Jade (from FG11, niche venue group), discussing a picture of her with friends posing with drinks in a hallway, was quick to stress that this was unusual behaviour associated with ‘big’ nights out or celebratory occasions, while Nadine (from FG5, niche venue group) justified her posed photos by explaining that it was due to contrasting gender norms in different friendship group (‘girly girls’):-

*Jade That*’*s a photo that my friend took before we went out … she would … take photos of everyone, really before we went out … cause it was New Year we were dressed up a bit more … just, my friends from home would be more, maybe more girls with me, so there would be a bit less like pulling ugly faces and stuff.*

*Nadine Like those kinds of photos; I don*’*t do it. I honestly don*’*t do that, it*’*s so stupid. But when I*’*m out with people from my work I do it because they*’*re all total girly girls and like to get dressed up and go on nights out.*

Although it might be disconcerting to be ‘caught’ with photos like this on a Facebook profile, the taking and posting of such images could be presented as having an ironic ‘knowing’ quality. For example, Kerry (from FG9, niche venue group) acknowledged in her Facebook interview that the ‘hall pose’ photo in her sparkly graduation dress was ‘embarrassing’ and ‘shameless’ but claimed that she ‘did it with awareness’:-

*Kerry This is like literally in a hall. You know, that*’*s really embarrassing. But this is the classic picture of girls standing, yeah, that*’*s exactly what it is. And it*’*s like shameless! (laughs). It*’*s just before my grad ball and I never like don*’*t really put on a dress that often. I don*’*t get glammed up and like big earrings and sparkly dresses and stuff like that…But yeah, it*’*s a classic picture. I did it with awareness like and. I*’*m gonna let myself off in this one cause I do think I look quite good.*

## Discussion

Our work reveals how the young women in this study attempted a difficult ‘balancing act’ both online and offline, demonstrating an ‘up for it’ sexy (but not too sexy) femininity through their drinking practices and appearance, while still retaining control and respectability (Griffin et al., 2013). While the concept of a gendered double standard around appearance and alcohol consumption is not new (Emslie et al., 2015), a wider online audience can now observe, judge and comment on how women look and behave (Manago et al., 2008), so continuous surveillance is required to construct successful and authentic self-presentations (Goodwin et al., 2016). This ‘balancing act’ was particularly precarious for working class women, who appeared to be judged more harshly (both off and on line) than middle class women.

Young women were aware of multiple audiences, and the value of the social currency their online content had to these different audiences. They worked extremely hard to create and share material which would be valued by close friends and wider acquaintances (Atkinson & Sumnall, 2016). Images in which young women perceived themselves to be overly intoxicated would not be uploaded to Facebook; this was not surprising given that women from a range of class positions used ‘positioned othering’ to distance themselves from ‘disgusting’ intoxicated women in public places (Hutton et al., 2016). Highly posed and controlled images were preferred by working class women. Their more limited economic, academic, symbolic and cultural capital may have constrained their freedom to adopt anything other than a traditional, emphasised, femininity. ‘Beauty capital’ was important for these working class young women, who were well aware of the market value of appearance (Huppatz, 2009); for some, their ‘true self’ was reflected in the glamorous images they posted online, rather than by their more mundane everyday presentations of self. In contrast, middle class women appeared to draw on a broader range of femininities in relation to alcohol related content online, striking a balance between apparently unposed ‘fun’ images which had value to close friends, and more formal, posed images to obtain value from a wider audience. They worked hard to draw boundaries between themselves and their working class peers, disparaging the latter’s ‘glamorous’ online images as inauthentic and fake.

While notions of ‘authenticity’ and ‘taste’ appear to be natural and normal, they are of course socially constructed. Thornton (1995) has developed Bourdieu’s theory of cultural capital (i.e. knowledge accumulated through upbringing and education which confers status) to develop the idea of ‘hipness’ as a form of subcultural capital which is objectified and embodied through consumption, style and attitude. Our data suggest that working class women accrued subcultural capital from both close friends and wider social networks through the online display of their ‘glamorous’ selves and were critical of women dressed too casually at venues where hypersexual performances were the norm. However, traditionally it is dominant groups who have the power to impose their definitions of taste on others, and decide what is ‘authentic’ and what is ‘fake’ (Bourdieu, 1984; Williams, 2006). The young middle class women in our study powerfully deployed their subcultural capital to contrast the ‘fake’ posed online images favoured by working class women with their own apparently unposed ‘authentic’ representations of their drinking occasions. While the individual interviews revealed that middle-class women still valued the posed, traditionally feminine representations of themselves “looking good”, they were able to draw on their subcultural capital to claim that they did this ‘with irony’ (also see Hutton, 2006). This process has striking parallels with Thornton’s clubbers from the 1990s, who contrasted their hip authenticity and ‘classless autonomy’ with the false and superficial, class-bound figures of ‘raving Sharon’ and ‘Techno Tracy’ (who had evolved from stereotypes of young working-class women wearing white high heels and miniskirts, drunkenly dancing round their handbags in mainstream discos). Thus, both online and offline, subcultural capital continues to be “a currency which correlates with and legimitizes unequal status” (Thornton, 1995) p104.

Middle class respondents (both female and male) demonstrated negative attitudes to working class women’s alcohol consumption. Similar drinking practices, such as drinking pints of lager or beer, were constructed as acceptable for themselves or for middle-class female friends but as stigmatizing for working class women. Photo prompts used in the focus groups which depicted dressed up women who appeared to be intoxicated were usually ‘read’ as representations of working-class women whose perceived lack of control was linked to a ‘spoiled’ sexual identity. Given that working class women struggle to attain ‘respectable’ femininity and have to work constantly to prevent a loss of reputation (Skeggs, 1997), it is perhaps not surprising that they were more wary than middle class women of posting drunken images online. The visceral disgust, or symbolic violence (Bourdieu, 1984) displayed by some middle class women towards what they read as embodied signifiers of social class (e.g. fake tan, certain shoes and clothing, particular facial features) was notable. Our findings echo McRobbie’s (2004) assertion that “feminine modalities of symbolic violence as processes of class differentiation are now thoroughly projected onto and inseparable from the female body” (p102). Respondents in our study also used coded references to class (Bailey et al., 2015) such as ‘ned’ (the Scottish equivalent of the English ‘chav’) and ‘council faced’ (the ‘look’ of a working class person living in local authority housing) to differentiate themselves from working class women, drawing on judgemental discourses from celebrity culture. Historically, fear and disgust have been used to position drunken working class women as posing a threat to society, resulting in further marginalisation for this already disadvantaged social group (Lupton, 2015). One way in which this marginalisation continues is through ‘chav talk’ (Shildrick, Blackman, & MacDonald, 2009) which links young, poor, white working-class people to welfare dependency, fecklessness and conspicuous consumption, and expresses underlying social anxieties (see also Atkinson & Sumnall, 2016). Shildrick et al conclude that “while offensive, prejudicial language rooted in other social divisions and disadvantage … has waned in popular discourse and all but disappeared in academic writing, class prejudice – in the form of ‘chav talk’ – proliferates in the UK” (p461).

This is the first UK study to explicitly explore both gender and social class in relation to the display of alcohol-related content online. A strength of our study was using both friendship-based discussion groups and individual Facebook interviews to collect data. This innovative methodological approach revealed differences between dominant discourses in the focus groups (e.g. middle class women mocking certain performances of femininity) and individuals displays of alcohol-related content online (e.g. photos of some participants engaged in behaviours which they had criticised in the focus groups), allowing us access to more nuanced accounts of gender performance.

Our study also has some limitations. Our findings are based on interview data from young people in one city in the west of Scotland. However, they are likely to have relevance beyond the Scottish context, given the parallels with findings from studies in the rest of the UK and New Zealand (Atkinson & Sumnall, 2016; Bailey et al., 2015; Lyons, Emslie et al., 2014; Lyons, McCreanor et al., 2014; Niland et al., 2014). Our data were collected between 2012 and 2013 and focused on Facebook, but there is little evidence to suggest that more recent studies of drinking practices using different social media would be very different. We did not directly observe young people’s drinking but this was because we were interested in exploring the meanings they attached to their drinking practices and their representations of alcohol online.

Our work adds to the limited literature which aims to explore how gender and social class play out in young people’s drinking cultures both offline and online. Lyons et al. (2016) have suggested that “technological practices around drinking and socialising reproduce older … regimes of power” (p11). Our findings are striking in the way they highlight this. Young women work hard to successfully negotiate gendered double standards around appearance and alcohol consumption to wider audiences as well as close friends. Further, working class women are constrained in the range of femininities from which they can draw, and judged particularly harshly, both online and offline. As such, our results might be dismissed as ‘nothing new’. However, we suggest their importance in demonstrating the continuing centrality of social structures such as gender and social class in the construction of identity in the age of social media.
